# Comprehensive Suppression of All Apoptosis-Induced Proliferation Pathways as a Proposed Approach to Colorectal Cancer Prevention and Therapy

**DOI:** 10.1371/journal.pone.0115068

**Published:** 2014-12-11

**Authors:** Michael Bordonaro, Eric Drago, Wafa Atamna, Darina L. Lazarova

**Affiliations:** 1 Department of Basic Sciences, The Commonwealth Medical College, 525 Pine Street, Scranton, PA 18509, United States of America; 2 California Northstate University, College of Medicine, 9700 West Taron Drive, Elk Grove, CA 95757, United States of America; Centro Cardiologico Monzino, Italy

## Abstract

Mutations in the WNT/beta-catenin pathway are present in the majority of all sporadic colorectal cancers (CRCs), and histone deacetylase inhibitors induce apoptosis in CRC cells with such mutations. This apoptosis is counteracted by (1) the signaling heterogeneity of CRC cell populations, and (2) the survival pathways induced by mitogens secreted from apoptotic cells. The phenomena of signaling heterogeneity and apoptosis-induced survival constitute the immediate mechanisms of resistance to histone deacetylase inhibitors, and probably other chemotherapeutic agents. We explored the strategy of augmenting CRC cell death by inhibiting all survival pathways induced by the pro-apoptotic agent LBH589, a histone deacetylase inhibitor: AKT, JAK/STAT, and ERK signaling. The apoptosis-enhancing ability of a cocktail of synthetic inhibitors of proliferation was compared to the effects of the natural product propolis. We utilized colorectal adenoma, drug-sensitive and drug-resistant colorectal carcinoma cells to evaluate the apoptotic potential of the combination treatments. The results suggest that an effective approach to CRC combination therapy is to combine apoptosis-inducing drugs (e.g., histone deacetylase inhibitors, such as LBH589) with agents that suppress all compensatory survival pathways induced during apoptosis (such as the cocktail of inhibitors of apoptosis-associated proliferation). The same paradigm can be applied to a CRC prevention approach, as the apoptotic effect of butyrate, a diet-derived histone deacetylase inhibitor, is augmented by other dietary agents that modulate survival pathways (e.g., propolis and coffee extract). Thus, dietary supplements composed by fermentable fiber, propolis, and coffee extract may effectively counteract neoplastic growth in the colon.

## Introduction

The improvement of anti-cancer preventive and therapeutic strategies has decreased cancer-related deaths by 20% in the past 20 years [1]. In addition, the concept of oncogene addiction [2] spurred the development of molecularly targeted therapies. However, most of these therapies extend the lives of cancer patients in average by a few months [3]. A reason for this outcome is that the neoplasms exhibit drug resistance mutations that are either pre-existent in a low number of cancer cells prior to treatment, or are acquired after drug administration [3]. In absence of resistance-conferring mutations, cancer cells also adapt to the selective drug pressure by adjusting their signaling levels. For example, *BRAF*-mutant melanoma cells exposed to vemurafenib develop resistance to the agent by upregulating their BRAF expression [4]. Similarly, EGFR-mutant lung cancer cells treated with an EGFR inhibitor downregulate PTEN and increase AKT survival signaling [5]. Therefore, the design of anti-cancer therapies should take into account not only the mutational landscape of a neoplasm, but also the cell signaling heterogeneity that exists even among genetically identical cancer cells [6]. The signaling heterogeneity is a component of the immediate mechanisms of resistance (IMR): these are the changes in signaling cascades that occur within 24 hours of treatment and allow a fraction of a cancer cell population to survive drug treatment.

CRC cells exposed to histone deacetylase inhibitors (HDACis) also exhibit IMR. We have provided evidence that HDACis induce apoptosis of CRC cells in part through hyperactivation of WNT/catenin signaling [7]. However, CRC cell populations are heterogeneous in terms of WNT/catenin levels, and cells that do not hyperinduce the pathway, survive exposure to HDACis [7], [8]. The signaling heterogeneity of cell populations is not due to the existence of cell subpopulations with pre-determined levels of WNT/catenin activity. Thus, we have flow cytometry-sorted single CRC cells with high WNT/catenin signaling levels, and found that the resulting clonal populations are as heterogeneous in WNT signaling levels as the parent population (data not published). This heterogeneity is likely maintained by lateral inhibition [9], an adoption of a particular fate by a few cells from a group of equivalent cells [10]. In this process, stochastic variations in the expression of the receptor NOTCH and its ligands on adjacent cells are amplified through cell-to-cell interactions. The interactions between NOTCH and its ligands lead to the release of their intracellular domains. The cells with higher levels of NOTCH intracellular domain suppress WNT activity; whereas, the cells with higher levels of NOTCH ligands increase WNT activity [9]. In normal intestinal cells, lateral inhibition contributes to terminal differentiation [11]; however, in CRC cells, lateral inhibition supports signaling heterogeneity without terminal differentiation. Similar signaling heterogeneity has been observed *in vivo* [12]–[15].

The second component of the IMR is the apoptosis-induced proliferation, a phenomenon supported by the apoptotic cells that secrete mitogens. These mitogens stimulate the proliferation of the surviving cells in any apoptotic population [16]–[21]. We have found that in CRC cell populations undergoing HDACi-triggered apoptosis, there is increased expression of mitogens and a subsequent induction of several survival signaling pathways [9], [22]. Here we report a strategy to inhibit comprehensively all survival pathways, and enhance the apoptotic response of CRC cells to both synthetic and diet-derived HDACis (e.g., LBH589 and butyrate).

## Materials and Methods

### 1. Cell culture, recombinant plasmids, and chemicals

The human CRC cell line HCT-116 was obtained from the American Type Culture Collection (Rockville, MD). HCT-116 cell line was genotyped by short tandem repeat analysis. The same analysis of HCT-R cells confirmed that these cells are related to the parental HCT-116 cells. The cell line HCT-R was derived from HCT-116 by culturing the parental cells in increasing concentrations of butyrate as previously described [8]. HCT-116 and HCT-R cell lines were grown in alpha-MEM medium with 10% fetal bovine serum (FBS). Human colonic microadenoma LT 97 cells were a kind gift from Dr. B. Marian (Institute of Cancer Research, Medical University Vienna, Austria). LT 97 cells were cultured as previously described [23], [24]. The following cell lines were cultured in alpha-MEM with 10% FBS: human small cell carcinoma adrenal gland/cortex SW13 cells (minus phenotype, isolated by dilution cloning from a heterogeneous sample ATCC CCL105 in the laboratory of Dr. Elizabeth Hull, Midwestern University, AZ), human neural crest-derived non-neuronal progenitor LA1-5s cells (provided by Dr. R. Ross, Fordham University, NY), normal human fetal colonic cells CCD841CoN ((ATCC CRL-1790), human embryonic kidney epithelial HEK293T cells (ATCC CRL-1573), and mouse embryonic fibroblasts CCL92 (ATCC CCL-92). Sodium butyrate was obtained from Sigma, caffeic acid phenethyl ester (CAPE)-enhanced propolis (Propolis with Cyclopower, with CAPE at 6 mg/g) from Manuka Health New Zealand Ltd, AZD6244, LBH589, and MK2206 from Selleck Chemicals, pyridone 6 from Santa Cruz Biotechnology, freeze dried instant coffee (The Daily Chef, Sam’s West, Inc.). All agents except butyrate were resuspended in dimethyl sulfoxide, and the stock solutions were kept at −80°C. Butyrate was dissolved in water and stored as 1.0 M stock at 4°C. Mock treatment included dimethyl sulfoxide in a volume equal to this of the treatments with agents dissolved in dimethyl sulfoxide.

### 2. Western blot analyses and antibodies

Western blot analyses were performed as reported previously [25]. The following antibodies were used: an antibody to phospho-p44/42 MAPK (Erk1/2) (Thr202/Tyr204) (#4370, Cell Signaling Technology), an antibody recognizing phospho-Stat3 (Tyr705) (#9145, Cell Signaling Technology), anti-Ser473-phosphorylated AKT (sc-7985, Santa Cruz Biotechnology), anti-AKT (sc-8312, Santa Cruz Biotechnology), anti-beta ACTIN (A5441, Sigma-Aldrich), anti- ERK (#9102, Cell Signaling Technology), anti- STAT3 (#9139, Cell Signaling Technology), anti-pcJUN and c-JUN (sc-16312-R and sc-45, Santa Cruz Biotechnology). All antibodies were utilized at working dilution of 1∶1,000, except the anti-ACTIN antibody that was utilized at 1∶5,000 dilution. Lysates were obtained via two different methods: by utilizing a sodium dodecyl sulfate -containing lysis buffer and quantifying the protein concentration [25], or by lysing equal number of cells (10^6^) directly in Laemmli buffer. Routinely, cells were plated a day prior to treatment, and exposed for 17 or 20 h with LBH589 (50 nM), MK2206 (1 µM), AZD6244 (0.5 µM), pyridone 6 (1 µM), coffee extract (1 mg/ml), propolis (100 µg/ml), or butyrate (5 mM). Quantification of the western blot images was performed with ImageJ software (public domain software developed by the Research Services Branch of the National Institute of Mental Health, Bethesda, MD, USA).

### 3. Apoptotic and clonal growth assays

CRC cells were plated 24 h prior to analyses in 24-well plates at 70,000 per well, and exposed to treatments for 24 hours (e.g., LT97 and HCT-116 cells) or 48 hours (HCT-R cells). All cells (floating and attached) were harvested and stained for apoptotic and necrotic markers with PE Annexin V Apoptosis Detection Kit I (BD Biosciences, #559763). Flow cytometry analyses were carried out with FACS Aria II and DiVa software; we analyzed total of 50,000 events per sample. Percent apoptosis was calculated by dividing the number of apoptotic cells by the total number of analyzed cells and multiplying the ratio by 100. For clonal growth analyses, single cells were plated at 100–200 cells per well in a six-well plate and treated with the agent(s) for 24 hours. Colonies were counted at 14 to 20 days after removal of the agent(s). All experiments were performed four to six times with triplicate samples per experiment.

### 4. Statistics

All data were presented as mean ± standard deviation from at least three sets of independent experiments. Unpaired student T-test analysis was used to determine the significance of statistical differences. Differences were considered significant at P<0.05. For clonal growth and apoptotic analyses, statistical differences between group means were determined by one-way analysis-of-variance (ANOVA) with GraphPad Prism 6 software, and the post-test calculations that used the Bonferroni correction to adjust for multiple comparisons with 95% confidence.

## Results

### 1. Development of inhibitor cocktail of apoptosis-induced proliferation (ICAP)

**Figure 1:**
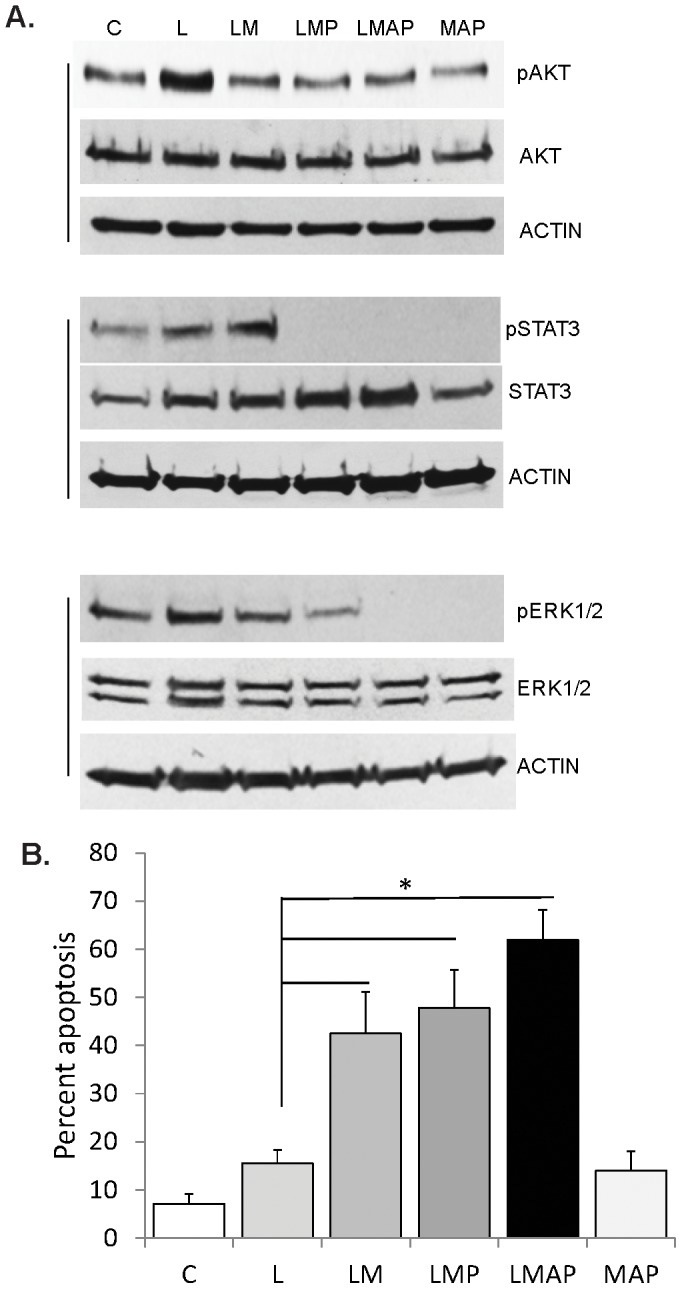
LBH589-induced apoptosis is augmented by suppressing three survival pathways. A. Western blot analyses of HCT-R CRC cells exposed to mock (C) treatment, 50 nM LBH589 (L), 1 µM MK2206 (M), 0.5 µM AZD6244 (A), or 1 µM pyridone 6 (P) for 20 hours. Equal number of cells were lysed directly in Laemmli buffer and analyzed for expression levels of phosphorylated and total levels of AKT, ERK1/2, and STAT3. B. Apoptotic analyses of HCT-R cells exposed for 48 h to the treatments described in (A). Asterisk indicates statistically significant differences (P<0.05) between the apoptotic levels. Additional statistically significant differences are indicated in the text.

We have demonstrated that HDACis induce apoptosis of CRC cells with mutations in the WNT/catenin pathway [7], and the levels of cell death are augmented by suppressing the induction of AKT signaling [22], [26]. To analyze all survival pathways induced in apoptotic CRC cell populations, we utilized HCT-R cells that are relatively HDACi-resistant compared to parental HCT-116 cells [8]. We determined the ability of an inhibitor cocktail of apoptosis-associated proliferation (ICAP) to augment apoptosis in CRC cell populations exposed to LBH589, a HDACi that is in clinical trials. To ascertain the clinical relevance of previous findings [22], [26], we employed pharmacologically relevant concentrations of LBH589 (50 nM) and the inhibitors of survival pathways [27], [28]. HCT-R cells exposed to LBH589 exhibited increased levels of three survival pathways mediated by phosphorylated AKT, STAT3 and ERK1/2 signaling molecules (Fig.1A). LBH589-treated cells exhibited 15.5±2.8% apoptosis, and the addition of MK2206, a pAKT inhibitor, almost tripled the levels of apoptosis to 42.5±8.6%, P = 0.007 (Fig.1B). This finding is in agreement with observations that suppressing pAKT levels by MK2206 or caffeic acid phenethyl ester augments apoptosis in CRC cells [22]. Addition of pyridone 6, a JAK/STAT inhibitor, and AZD6244, an adenosine triphosphate-uncompetitive inhibitor of MEK1/2 [29], [30] increased apoptosis further. Exposure to the ICAP alone resulted in apoptosis of 14.0±4.0%, compared to 7.1±2.0% apoptosis induced by mock treatment. Ordinary one-way ANOVA revealed statistically significant differences between group means, F(5,12) = 38.56, P<0.0001. Post-test calculations with Bonferroni correction to adjust for multiple comparisons with 95% confidence indicated statistically significant differences (P<0.05) in the apoptotic levels between mock treatment and all three combination treatments with LBH589, as well as between LBH589 treatment and all three combination treatments with LBH589. There were no statistically significant differences between the apoptotic levels of mock-, LBH589-, and ICAP - treated cells. There were no statistically significant differences in apoptosis induced by LBH589 + MK2206 and the other two combination LBH589 treatments, as well as between LBH589 + MK2206 + pyridone6 and LBH589 + ICAP.

### 2. Propolis augments the apoptosis induced by LBH589 or 5-fluorouracil in CRC cells

**Figure 2:**
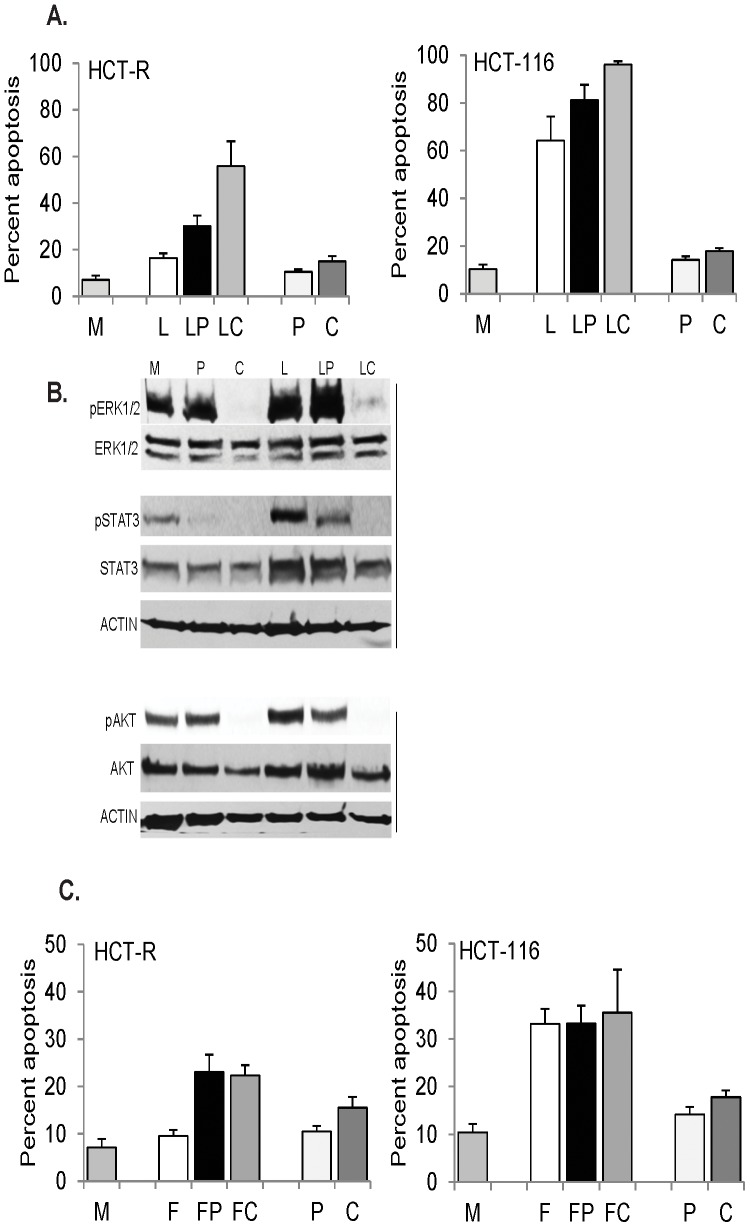
Ability of ICAP and propolis to augment chemotherapeutics-induced apoptosis. A. HCT-R or HCT-116 cells were exposed for 48 h or 24 h respectively, to mock (M), 50 nM LBH589 (L), LBH589 and 100 µg/ml propolis (LP), LBH589 and the ICAP (LC), 100 µg/ml propolis (P), or the ICAP alone (C). B. HCT-R cells were exposed to the treatments described in A for 17 h. and total cell lysates were analyzed by western blotting. C. HCT-R and HCT-116 cells were exposed for 48 h or 24 h respectively, to mock (M), 10 µM 5-fluorouracil (F), 5-fluorouracil and 100 µg/ml propolis (FP), 5-fluorouracil and the ICAP (FC), propolis (P), or the ICAP alone (C).

Propolis, a honeybee product, augments apoptosis induced by the diet-derived HDACi butyrate via partial suppression of AKT and JAK/STAT signaling [26]. Therefore, we reasoned that propolis may augment LBH589-induced apoptosis via the same mechanism. To compare the ability of propolis and the ICAP to suppress apoptosis-induced survival pathways, we exposed HDACi-resistant HCT-R cells to mock treatment, LBH589, LBH589 + propolis, or LBH589 + ICAP. Addition of propolis augmented LBH589-induced apoptosis from 16.5±2.0% to 30.1±4.5%, P = 0.013 (Fig.2A); whereas, addition of the ICAP enhanced LBH589-induced apoptosis from 16.5±2.0% to 60.0±10.6%, P = 0.004 (Fig.2A). Ordinary one-way ANOVA revealed statistically significant differences between all group means, F(5,12) = 39.35, P<0.0001. Post-test calculations with Bonferroni correction to adjust for multiple comparisons with 95% confidence revealed statistically significant differences (P<0.05) in the apoptotic levels between mock treatment and the two LBH589 combination treatments, but not LBH589 alone. Significant differences were also noted for the apoptotic levels induced by LBH589 and LBH589 + ICAP, as well as by LBH589 + propolis and LBH589 + ICAP. There was no statistically significant difference between the apoptosis of cells exposed to mock, propolis, or ICAP treatment. Western blot analyses revealed that in LBH589-treated HCT-R cells, co-treatment with propolis decreased pSTAT3 levels by six-fold and pAKT levels by 1.7-fold; whereas, co-treatment with ICAP resulted in undetectable levels of both pSTAT3 and pAKT (Fig.2B). The most striking difference between the two combination treatments with LBH589 was that compared to the exposure to LBH589 alone, addition of propolis increased the pERK1/2 levels by 20%; whereas, ICAP decreased pERK1/2 levels by eight-fold (Fig.2B).

HCT-R cells are relatively resistant to the apoptotic effects of HDACis in part due to their upregulated survival signaling even in absence of treatment [8], [22]. To evaluate the significance of the survival pathways in HDACi-sensitive CRC cells, we utilized HCT-116 cells, from which HCT-R cells were derived [8]. In HCT-116 cells, treatment with LBH589 alone resulted in a six-fold increase of apoptosis (mock treatment resulted in 10.4±1.8% apoptosis, and LBH589 exposure at 50 nM led to 64.2±10.0% apoptosis, P = 0, Fig.2A). Addition of propolis or the ICAP augmented further the apoptotic effect of LBH589: the exposure to LBH589 + propolis resulted in 81.3±6.3% apoptosis (P = 0.072), and LBH589 + ICAP led to 96.0±1.5% apoptosis (P = 0.006), Fig.2A. One-way ANOVA revealed statistically significant differences between group means, F(5,12) = 162.1, P<0.0001. Post-test calculations with Bonferroni indicated statistically significant differences (P<0.05) in the apoptotic levels between mock treatment and all three treatments that included LBH589. The same significant differences were noted for the apoptotic levels induced by propolis or ICAP, and all three treatments that included LBH589. There was no statistically significant difference between the apoptotic levels of cells exposed to mock, propolis, or ICAP treatment.

LBH589 is highly effective in inducing apoptosis of CRC cells with mutations in the WNT/catenin pathway; however, the agent is still in clinical trials. Therefore, we asked whether propolis and the ICAP augment apoptosis induced by 5-fluorouracil, a major component of current anti-CRC therapeutic regimens. 5-Fluorouracil (5-FU) induces low levels of apoptosis in HCT-116 cells at the unachievable *in vivo* concentration of 10 µM (mock cells exhibit 10.4±1.8% apoptosis, and 5-FU-treated cells – 33.1±3.2% apoptosis, P = 0, Fig.2C. At the same concentration the agent does not induce significant apoptosis in HCT-R cells: mock-treated cells exhibit 7.1±1.8% apoptosis, and 5-FU-treated cells - 9.5±1.3% apoptosis, P = 0.14, Fig.2C. The combined exposure of HCT-116 cells to 5-FU + propolis or 5-FU + ICAP did not increase significantly the apoptotic levels compared to the treatment with 5-FU alone: 5-FU exposure resulted in 33.1±3.2% apoptosis, 5-FU + propolis treatment led to 33.2±3.8% apoptosis, and 5-FU + ICAP - to 35.5±9.0% apoptosis (Fig.2C). Ordinary one-way ANOVA revealed statistically significant differences between group means, F(5,12) = 18.31, P<0.0001. Post-test calculations with Bonferroni correction to adjust for multiple comparisons with 95% confidence indicated statistically significant differences (P<0.05) in the apoptotic levels between mock treatment and all three treatments that included 5-FU. The same significant difference was observed for the apoptotic levels induced by propolis or ICAP compared to all three treatments that included 5-FU.

Both propolis and ICAP doubled the apoptotic response of HCT-R cells to 5-FU: compared to 5-FU alone, 5-FU + propolis treatment led to 23.1±3.6% apoptosis, P = 0.003, and 5-FU + ICAP – 22.3±2.2% apoptosis, P = 0.001, Fig.2C. One-way ANOVA of apoptotic levels of HCT-R cells exposed to combination treatments with 5-FU and ICAP or propolis revealed statistically significant differences between group means: F(5,12) = 28.81, P<0.0001. Post-test calculations with Bonferroni correction indicated statistically significant differences (P<0.05) in the apoptotic levels between mock treatment and 5-FU + propolis, between mock treatment and 5-FU + ICAP, and between 5-FU treatment and the combination treatments of 5-FU + propolis and 5-FU + ICAP. There was no statistically significant difference between the apoptotic levels of mock and 5-FU treatment.

### 3. Targeting apoptosis-associated proliferation as a colon cancer preventive approach

**Figure 3:**
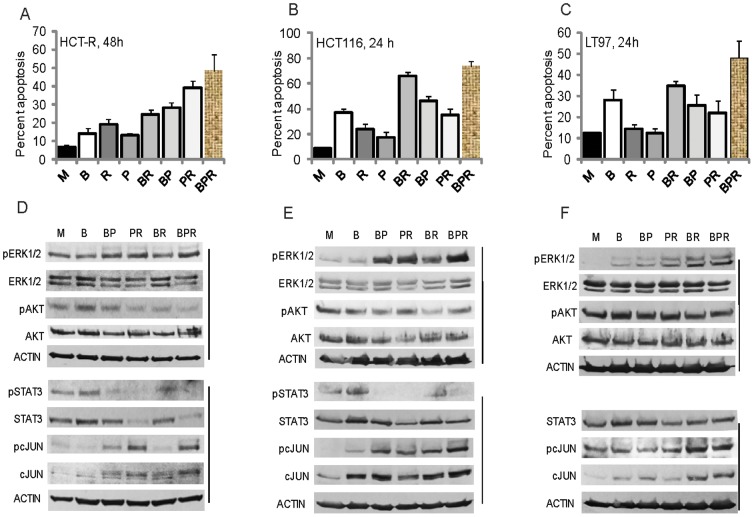
The combination treatment of butyrate, propolis, and coffee extract induces highest levels of apoptosis in colon adenoma and carcinoma cells. A–C. Cells were exposed to mock (M), 5 mM butyrate (B), 1 mg/ml roasted coffee extract (R), 100 µg/ml propolis (P), butyrate and coffee extract (BR), butyrate and propolis (BP), propolis and coffee extract (PR), or butyrate, propolis and coffee extract (BPR). Apoptosis was measured by flow cytometry as described in Materials and Methods. D–F. Cells were exposed to treatments as described above for 17 h, and total cell lysates were analyzed by western blotting.

Whereas the clinical application of the ICAP and a propolis supplement to augment anti-cancer therapies will require validation through randomized clinical trials, the application of a diet-based supplement in CRC prevention is more tangible. Butyrate, a fermentation product of fiber in the colon and a HDACi, induces apoptosis in most CRC cell lines, and this effect may explain in part the protective role of fiber against CRC [31]. Similar to the apoptosis induced by LBH589, apoptosis initiated by butyrate is counteracted by survival signaling. Therefore, a CRC preventive approach may combine butyrate with inhibitors of the survival pathways. Propolis augments butyrate-induced apoptosis by suppressing two survival pathways: AKT and JAK/STAT [26]; however, in our analyses of LBH589-treated CRC cells, propolis not only did not suppress, but in fact, augmented pERK1/2 levels. Since the suppression of ERK signaling by the MEK1/2 inhibitor AZD6244 enhanced the apoptotic effect of LBH589 (Fig.1B), we reasoned that butyrate/propolis-induced apoptosis could be similarly augmented by targeting ERK signaling with diet-derived compounds. Based upon a literature search, we focused on several reported diet-derived ERK1/2 inhibitors, among which were ursolic acid, curcumin, sulforaphane, and coffee. From the screened compounds none suppressed pERK1/2 levels in butyrate/propolis-treated CRC cells; however, addition of coffee extract enhanced apoptosis of HCT-R cells (Fig.3A). One-way ANOVA revealed statistically significant differences between the group means: F(7,25) = 50.96, P<0.0001, (Fig.3A). Post-analyses with Bonferroni correction were performed for all eight groups of treatments; however, we focused on whether adding coffee extract to previously characterized and reported by us dietary agents influenced apoptotic levels of HCT-R cells. Thus, we found that there were statistically significant differences (P<0.05) in the apoptotic levels between the following treatments: butyrate versus butyrate+coffee, butyrate+propolis versus butyrate+propolis+coffee, and propolis versus propolis+coffee. The two regimens that induced the highest levels of apoptosis in HCT-R cells, propolis+coffee and butyrate+propolis+coffee, reduced the pSTAT3 to undetectable levels and pAKT levels by three-fold (Figs.3A and 3D).

HCT-R cells likely represent the late stages of neoplastic development, when drug resistance is established. However, it is more relevant to test any CRC preventive strategy in cells that represent earlier neoplastic stages. Therefore, we analyzed the effects of diet-derived agents in the LT97 cell line established from a human colonic microadnoma [23], [24], and HCT-116 colon carcinoma cells that are sensitive to chemotherapeutic drugs. Compared to HCT-R cells, both LT97 and HCT-116 cell lines are more sensitive to butyrate-induced apoptosis; therefore, we measured apoptosis at 24 hours after the administration of different regimens. In HCT-116 cells, butyrate treatment resulted in 36.8±3.4% apoptosis compared to mock cells that exhibited 8.8±0.4% apoptosis (Fig.3B). The highest levels of apoptosis were achieved by the combinations of butyrate + propolis (46.0±2.0%), butyrate + coffee (65.8±3.5%), and butyrate + propolis + coffee (73.9±3.7%); the same combinations of agents also suppressed the levels of pSTAT3 (Fig.3E). One-way ANOVA for the levels of apoptosis of HCT-116 cells exposed to dietary agents revealed statistically significant differences between the group means: F(7,23) = 168.7, P<0.0001. Post-test calculations with Bonferroni correction to adjust for multiple comparisons with 95% confidence indicated statistically significant differences (P<0.05) in the apoptotic levels between all treatments except for butyrate treatment compared to butyrate + propolis, butyrate treatment compared to propolis + coffee, and butyrate + coffee treatment compared to butyrate + coffee + propolis.

We have previously established that LT97 cells are highly sensitive to WNT signaling hyperactivation and cell growth arrest in the presence of butyrate [32]; in these cells, mock treatment results in 12.2±0.7% apoptosis, and butyrate exposure in 27.8±5.0% apoptosis. Exposure of LT97 cells to propolis + coffee or propolis + butyrate resulted in statistically comparable levels of apoptosis: 22.1±4.7% and 25.6±2.1%, respectively. The highest levels of apoptosis were achieved by the combination treatments butyrate + coffee (34.7±5.7%) and butyrate + coffee + propolis (47.9±8.2%), and there was no statistically significant difference in the apoptotic levels achieved by the two treatments (P = 0.08). One-Way ANOVA for the levels of apoptosis of LT97 cells exposed to dietary agents revealed statistically significant differences between the group means: F(7,16) = 22.57, P<0.0001. Post-test analyses of the treatments that included coffee extract indicated that there were no statistically significant differences between the apoptotic levels of cells exposed to butyrate versus butyrate + coffee, and propolis versus propolis + coffee; however, there was a statistically significant difference between the apoptosis induced by butyrate + propolis and butyrate + propolis + coffee (P<0.05). Under the conditions used, we were unable to detect pSTAT3 levels in LT97 cells; however, exposure to butyrate + propolis + coffee extract increased pERK to detectable levels compared to mock-treated cells (Fig.3F). Similarly, exposure of HCT-116 cells to butyrate + propolis + coffee extract increased pERK levels by two-fold; however, such effect was not observed in HCT-R cells (Figs.3A, B). We also analyzed the levels of pc-JUN, as this pathway could affect apoptosis and cell survival. In all cells, total c-JUN levels increased with treatments, and pc-JUN levels reflected closely this increase (Figs.3D, E, F).

**Figure 4:**
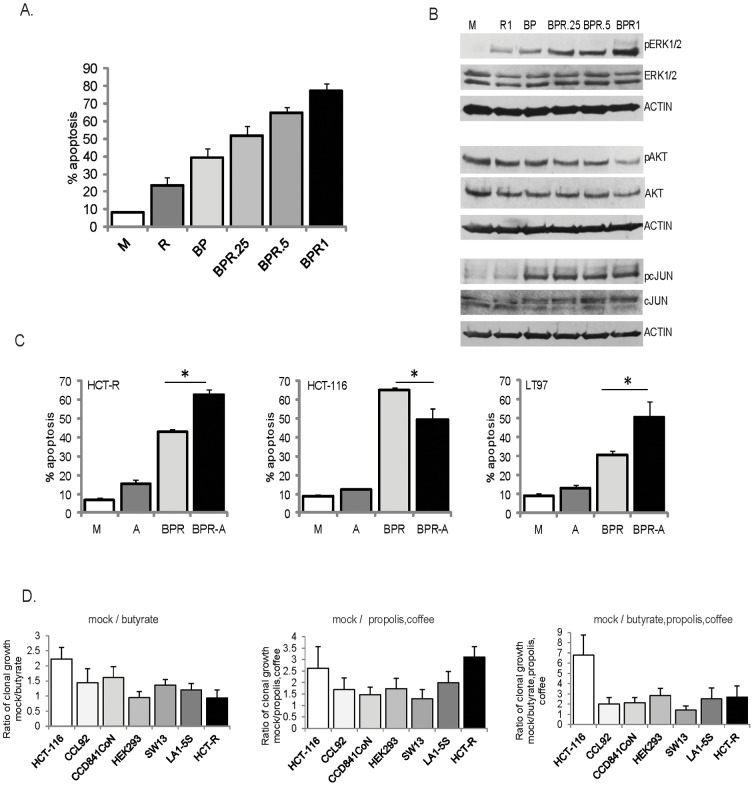
Role for ERK signaling in the apoptosis of CRC cells exposed to dietary agents and clonal growth ability of neoplastic and normal cells exposed to such agents. A. HCT116 cells were exposed for 24 h to mock (M), 1 mg/ml roasted coffee extract (R), 5 mM butyrate and 100 µg/ml propolis (BP), or butyrate/propolis and increasing concentrations of coffee extract: 0.25, 0.5 or 1.0 mg/ml. Apoptosis was measured by flow cytometry as described in Materials and Methods. B. HCT-116 cells were treated as in (A) for 17 hours and total cell lysates were analyzed by western blotting. C. HCT-116 and LT 97 cells were exposed for 20 h, and HCT-R cells were exposed for 42 h to mock (M), 0.5 µM AZD6244 (A), the combination; 5 mM butyrate, 100 µg/ml propolis, 1 mg/ml roasted coffee extract (BPR), or BPR and 0.5 µM AZD6244 (BPR-A). Apoptosis was measured by flow cytometry, as described in Materials and Methods. Statistically significant differences in apoptotic levels are noted by asterisks (P<0.05). D. Clonal growth assays. Percent clonal growth was calculated by dividing the number of cell colonies by the number of plated cells, and multiplying by 100. The ratios of clonal growth were calculated by dividing the percent clonal growth in mock-treated samples by the percent clonal growth in agent-treated samples. Experiments were repeated four to six times with triplicate samples per experiment. Statistically significant differences among cell lines in their mean ratios were determined by one-way ANOVA. For cells exposed to butyrate, F(6,31) = 10.26, P<0.0001; for cells exposed to propolis and coffee extract, F(6,28) = 5.493, P = 0.0007, and for cells exposed to butyrate, propolis and coffee extract, F(6,26) = 10.56, P<0.0001. The post-test calculations used the Bonferroni correction to adjust for multiple comparisons with 95% confidence. Among the cells exposed to butyrate, statistically significant differences (P<0.05, CI 95%) were detected in HCT116 vs. CCL92, HCT116 vs. HEK293, HCT116 vs. SW13, HCT116 vs. LA1-5s, HCT116 vs. HCT-R, CCD841CoN vs. HEK293, and CCD841CoN vs. HCT-R cells. Among the cell lines exposed to propolis and coffee extract, statistically significant differences (P<0.05, CI 95%) were detected in HCT116 vs. SW13, HCT-R vs. CCL92, CCD841CoN vs. HCT-R cells, HEK293 vs. HCT-R, and SW13 vs. HCT-R cells. Among cell lines exposed to butyrate, propolis, and coffee extract statistically significant differences (P<0.05, CI 95%) were detected in HEK293 vs. SW13 cells, and HCT116 vs. all other cell lines except for HCT-R cell line.

To test the effect of coffee at different levels, we exposed HCT-116 cells to 0.25, 0.5, and 1 mg of coffee extract per milliliter medium. The high dose of one milligram per milliliter was based upon coffee intake studies with ileostomy volunteers [33], and the average combined volume of fluids in stomach, small intestine, and colon [34]. At 1 mg/ml, coffee extract resulted in 23.7±4.6% apoptosis compared to mock treatment, 8.4±0.4% apoptosis (P = 0.001), Fig.4A. Treatment of the cells with butyrate + propolis resulted in 39.3±5.1% apoptosis, and the addition of 0.25, 0.5, or 1.0 mg/ml coffee extract increased apoptosis to 51.8±5.7% (P = 0.017), 64.8±3.2% (P = 0), or 77.4±4.1% (P = 0), respectively, Fig.4A.

Since increasing levels of coffee extract resulted in increasing levels of pERK1/2 and pc-JUN (Figs.3 and 4B), we analyzed the contribution of these signaling molecules to the apoptotic levels in the cells. Suppression of pc-JUN with SP600125, an inhibitor of the upstream JNK enzymes, did not result in significant changes in the apoptotic levels of cells exposed to butyrate + propolis + coffee extract (data not shown). Addition of the MEK1/2 inhibitor AZD6244 resulted in decreased apoptosis in HCT-116 cells from 65.0±1.6 to 49.4±5.7, P = 0.001, Fig.4C. However, treatment with AZD6244 increased apoptosis in LT97 (from 30.8±1.9% to 50.8±7.9%, P = 0.013), and HCT-R cells (from 42.7±1.7% to 62.7±2.41%, P = 0), Fig.4C.

To assess the long-term effect of exposure to the three diet-derived agents, we performed clonal growth assays (Fig.4D). LT97 cells do not re-grow from single-cell suspensions (data not shown); therefore, we compared the clonal growth ability of HCT-116 and HCT-R cells to this of five additional normal and neoplastic cell lines: small cell carcinoma adrenal gland/cortex SW13 cells, neuroblastoma LA1-5s cells, normal fetal colonic cells CCD841CoN, embryonic kidney epithelial HEK293T cells and normal embryonic fibroblasts CCL92. In preliminary experiments with increasing levels of butyrate, propolis and coffee extract we established the treatment concentrations that allow for 20% clonal growth of HCT-116 cells after a 24-hour exposure (i.e., 2 mM butyrate, 25 µg/ml propolis, and 0.2 mg/ml coffee extract), and used this combination treatment in all clonal growth assays. All cell lines exhibited differential sensitivity to butyrate, and the ability of butyrate to suppress clonal growth decreased in the following order: HCT116> CCD841CoN> CCL92> SW13> LA1-5S> HEK293  =  HCT-R. The ability of propolis and coffee extract to suppress clonal growth decreased in the following order: HCT-R> HCT116> LA1-5s> HEK293  =  CCL92> SW13> CCD841CoN. Exposure to all three diet-derived agents, butyrate, propolis and coffee extract, suppressed clonal growth in the following order of decreasing response: HCT116> HEK293> LA1-5S  =  HCT-R> CCL92  =  CCD841 CoN> SW13.

## Discussion

Targeting apoptosis-induced survival signaling as an approach to augment cancer therapies has been previously discussed [35]. ERK signaling has been identified as one target in augmenting cancer therapy, and the inhibition of the upstream MEK1/2 enhances the effect of some chemotherapeutics [36]. Numerous studies have focused on a single survival pathway as an approach to enhance anti-cancer therapies; however, it is known that the inhibition of one survival pathway leads to the compensatory activation of additional protective pathways [37]. This observation is supported by our findings that the suppression of AKT signaling in LBH589-exposed CRC cells induces higher pSTAT3 levels (Fig.1A). This redundancy in survival signaling is a reason to use a combination of inhibitors to counteract all apoptosis-induced survival. We have ascertained the ability of an inhibitory cocktail of apoptosis-associated proliferation (ICAP) to augment the HDACi-induced apoptosis of CRC cells. The ICAP was composed by inhibitors of pAKT, pERK1/2 and pSTAT3, and its use resulted in a three-fold increase in the apoptosis of the drug-resistant HCT-R cells, and a 1.5-fold increase in apoptosis in HDACi-sensitive HCT-116 cells. Therefore, incorporating the ICAP in HDACi-based therapies may specifically enhance the effect in drug-resistant cancer patients.

Based upon our previous studies [26], we posited that similar to the ICAP, propolis containing caffeic acid phenethyl ester (such as Propolis with Cyclopower, Manuka Health New Zealand Ltd) augments anti-cancer therapeutics by suppressing apoptosis-initiated survival signaling. Comparative analyses revealed that Propolis with Cyclopower suppresses pSTAT3 and pAKT levels; however, it does not suppress pERK1/2 levels in LBH589-treated CRC cells (Fig.2). In HCT-R cells, adding propolis or the ICAP to LBH589 treatment for 48 h resulted in statistically significant increase in apoptosis (30.1±4.5% and 55.9±10.6%, respectively). In HCT116 cells, adding propolis or the ICAP to LBH589 enhanced apoptosis to different extent, with the ICAP co-treatment resulting in a statistically significant increase in cell death (Fig.2A).

We also investigated the role of the apoptosis-associated survival in CRC cells exposed to 5-FU. We analyzed 5-FU at 10 µM due to the inability of the agent to induce apoptosis of CRC cells at lower concentrations. The addition of propolis or ICAP to 5-FU did not increase apoptosis of HCT-116 cells; whereas, the two co-treatments equally re-sensitized the drug-resistant HCT-R cells to the apoptotic effect of 5-FU (Fig.2C). These results are concordant with reports that Croatian propolis prolongs the suppressive effect of 5-FU on 4T1 mammary carcinoma [38], and oral administration of propolis with 5-FU or mitomycin C increases tumor regression [39].

We utilized the MEK inhibitor AZD6244 and the AKT inhibitor MK2206 at their maximally achievable *in vivo* concentrations [27], [28]; however, it is unknown whether the combined toxicity of the agents would allow achieving the same levels *in vivo*. Pyridone 6, the JAK/STAT inhibitor included in the ICAP, is not used *in vivo*; however, there are clinically administered JAK/STAT inhibitors that could substitute for pyridone 6. Whereas the ICAP is effective in augmenting HDACi-induced apoptosis of CRC cells *in vitro* (Figs.1 and 2), the clinical application of the ICAP is challenging. Guidance on how to co-develop two or more drugs in combination has been published by the Food and Drug Administration [40]; however, scientific and regulatory issues are a hurdle. As a result, although the ICAP is effective in augmenting the apoptotic effect of HDACis, and probably that of other chemotherapeutics, its immediate clinical application is unlikely. The utility of our findings is in demonstrating the concept of augmenting therapies through the inhibition of apoptosis-induced survival signaling. This paradigm can guide not only therapeutic, but also cancer-preventive strategies. In our search for effective diet-based CRC prevention, we found that propolis and coffee extract enhance the apoptotic effect of the dietary fiber-derived HDACi butyrate (Fig.3). Our *in vitro* data may explain the reported protective effect of coffee against CRC [41], [42]. Contrary to reports that coffee ingredients or metabolites suppress ERK signaling [43], [44], under our experimental conditions, coffee extract did not suppress pERK1/2 levels in butyrate/propolis-treated CRC cells (Figs.3,4). Unlike the other natural reagents used in our study, the coffee extract was prepared from a consumer-available coffee that is not standardized in terms of active components. The three major active components of coffee are caffeine, caffeic acid, and chlorogenic acid; however, there are numerous other components that may have biological activity [45]. Which of these coffee components influence the physiology of CRC cells will be the subject of future studies.

Our results support a pro-apoptotic role for pERK1/2 in HCT-116 cells and an anti-apoptotic effect of pERK1/2 in LT97 and HCT-R cells (Fig.4C). In addition to supporting proliferation, ERK signaling could mediate apoptosis when ERK activation is sustained [46]. Therefore, the significant increase in pERK levels in HCT-116 cells exposed to butyrate + propolis + coffee (Fig.4A) may contribute to the apoptotic outcome (Fig.4C). In butyrate + propolis + coffee-treated HCT-R and LT97 cells the moderate change in pERK levels may explain its anti-apoptotic role (Fig.4C).

From the perspective of formulating a CRC preventive strategy, the effects of diet-derived agents on microadenoma LT97 cells are of most interest. In these cells, representative of the earliest neoplastic lesions [23], [24], among the singly-administered agents, butyrate had the highest apoptotic effect. The addition of propolis and coffee augmented apoptosis, most likely by suppressing pAKT levels (Fig.3C). We were unable to detect pSTAT3 levels in LT97 cells; however the levels of total STAT3 were decreased by the combination treatments (Fig.3C). The sensitivity of early adenoma cells to the apoptotic effect of butyrate (Fig.3) is in agreement with the upgraded protective effect of dietary fiber against colon cancer from “probable” to “convincing” [31]. Despite the evidence for the CRC protective effect of fiber, it is difficult to impose a high fiber diet on the general population. Our results suggest that the combination of propolis and coffee may equal the protective ability of butyrate and therefore, a propolis + coffee supplement may substitute for high-fiber diet. Future experiments with primary neoplastic cells and animal models of intestinal cancer should be utilized to validate the reported here *in vitro* data. Promising results from such studies will support clinical trials with dietary interventions in populations with high-risk for CRC.

The ability for clonal growth of all six cell lines was decreased most significantly by the combined exposure to butyrate, propolis, and coffee extract (Fig.4D). The three cell lines with the least affected clonal growth were the small cell carcinoma adrenal gland/cortex SW13 cells, the normal mouse fibroblasts CCL92, and normal human fetal colonic CCD841CoN cells. The differential apoptotic levels and/or clonal growth ability of HCT116, LT97, and HCT-R exposed to the three diet-derived agents may explain why epidemiologic studies on the association between diet and cancer risk have yielded mixed results [47]. Thus, similar to the differential response of cancer cells to therapeutics, neoplastic cells respond differentially to diet-derived agents.

**Figure 5:**
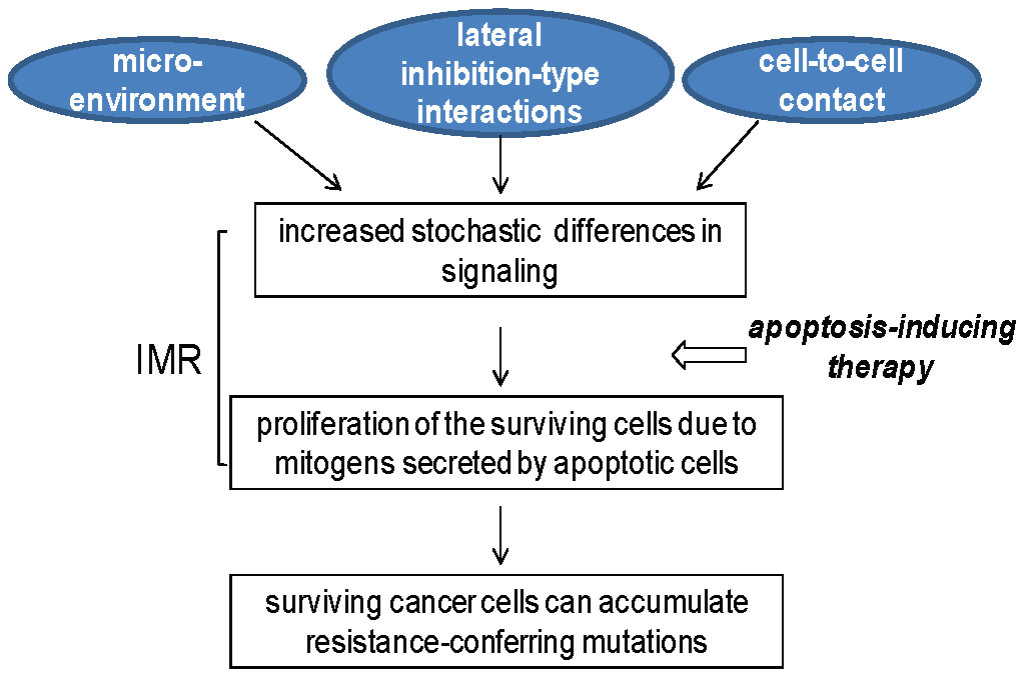
Hypothetical mechanisms of the immediate resistance (IMR) that facilitate survival of CRC cells in the presence of HDACis. Stochastic differences in signaling levels are always present in cell populations, and these differences are augmented by lateral inhibition-type interactions, microenvironment, and exposure to apoptotic agents [7], [12]–[15]. Due to their different signaling levels (in particular, variability in WNT/beta-catenin activity), not all CRC cells commit to apoptosis within 24 hours of exposure to HDACis. Apoptosis-induced mitogens [9], [22] may allow for the survival of a limited number of cells; in *in vivo* conditions, such cells could accumulate resistance-conferring mutations in time.

In summary, we hypothesize that two phenomena intrinsic to cell populations contribute to IMR: cell signaling heterogeneity, supported by a process similar to that of lateral inhibition, and apoptosis-induced survival (Fig.5). Consequently, an approach to CRC combination therapy should be composed by two modules: apoptosis-inducing drugs and agents that suppress apoptosis-induced survival. The same concept can be applied to CRC prevention, as the apoptotic effect of butyrate, a diet-derived HDACi, is augmented by dietary agents that modulate survival pathways.
